# Interpretation of Drain Amylase After Esophagectomy: A Case of Pancreatitis Mimicking Anastomotic Leak

**DOI:** 10.1016/j.atssr.2026.02.021

**Published:** 2026-03-05

**Authors:** Dena Shehata, Elliot L. Servais

**Affiliations:** 1 Division of Thoracic and Cardiovascular Surgery, Lahey Hospital and Medical Center, Burlington, Massachusetts; 2 UMass Chan–Lahey School of Medicine, Burlington, Massachusetts

## Abstract

Elevated pleural amylase is widely used as a sensitive, noninvasive screen for anastomotic leak after esophagectomy. We describe a 71-year-old man with mild rise in pleural amylase on postoperative day 7 despite a normal esophagram and clinical status. Elevated serum amylase and lipase confirmed acute pancreatitis as the source, rather than an anastomotic complication. Serum and drain amylase levels normalized with supportive care. This case demonstrates that modest elevations in pleural drain amylase, although highly useful for early leak detection, may occasionally represent false positives from alternative processes such as pancreatitis, underscoring the importance of an evaluation for nonanastomotic causes.

Early detection of intrathoracic anastomotic leak after esophagectomy remains essential because of the risk of high morbidity and mortality with delayed diagnosis. Monitoring pleural drain amylase has emerged as a sensitive and noninvasive adjunctive tool in this context.^1^, ^2^, ^3^

Multiple studies have demonstrated that elevated drain amylase correlates with anastomotic leak after esophagectomy.^1^, ^2^, ^3^, ^4^ A retrospective series found that drain amylase measurement had higher sensitivity for leak detection than contrast esophagram in the early postoperative period.^2^ In another cohort of patients undergoing Ivor Lewis esophagectomy, significantly higher drain amylase levels were observed in leak vs nonleak patients.^3^ A more recent study showed that serial pleural fluid amylase sampling, when combined with inflammatory markers such as serum C-reactive protein, may allow earlier and more accurate prediction of esophagogastric anastomotic leakage than either modality alone.^4^

Although drain amylase is a highly sensitive indicator, it is not entirely specific to the esophageal anastomosis.^5^ Elevated pleural fluid amylase can arise from pancreatic disease (eg, pancreatitis, pancreaticopleural fistula), malignant neoplasia, or other causes.^5^ In the setting of early or low-grade pancreatic disease, pleural or mediastinal fluid may become heavily loaded with pancreatic-type amylase and be manifested with minimal or absent abdominal signs or symptoms.

This report serves to emphasize that a mild or unexpected rise in drain amylase after esophagectomy, in an otherwise reassuring postoperative course, should prompt evaluation for pancreatitis as a rare but important potential confounder.

A 71-year-old man with biopsy-proven distal esophageal adenocarcinoma underwent staging and multidisciplinary evaluation. He completed 4 cycles of neoadjuvant chemotherapy with the FLOT regimen (fluorouracil, leucovorin, oxaliplatin, and docetaxel), which he tolerated well. Restaging with positron emission tomography/computed tomography demonstrated a robust tumor response. After detailed preoperative counseling, the patient was scheduled to undergo a standard Ivor Lewis esophagogastrectomy with intrathoracic anastomosis.

The operation proceeded without intraoperative complications. During the first 4 days postoperatively, his course remained stable; on postoperative day (POD) 4, the nasogastric tube was removed, and he continued on maintenance fluids and gradual mobilization. Pleural drain amylase was checked daily starting on POD 2, per our institutional protocol, and remained low and stable initially.

On POD 5, a contrast esophagram was performed, revealing a normal-appearing intrathoracic esophagogastric anastomosis without evidence of leak. The patient gradually advanced from sips to a full liquid diet during the next 2 days. On POD 7, routine analysis of the pleural drain fluid revealed a marked increase in amylase (Figure), raising concern for a possible occult anastomotic leak. However, the patient remained clinically stable; he was afebrile and hemodynamically normal, without leukocytosis or signs of sepsis or systemic inflammatory response. Given the unexpected rise in drain amylase, additional laboratory studies were obtained; serum amylase returned at 1340 U/L, and serum lipase was 120 U/L, consistent with acute pancreatitis. The patient was made NPO, prescribed intravenous fluid therapy, and closely monitored. A right upper quadrant ultrasound was performed that did not identify any abnormalities. During the next several days, the patient’s serum and drain amylase levels normalized. He was advance to a liquid diet, the pleural drain was removed, and he was discharged home on POD 11.

**Figure.**
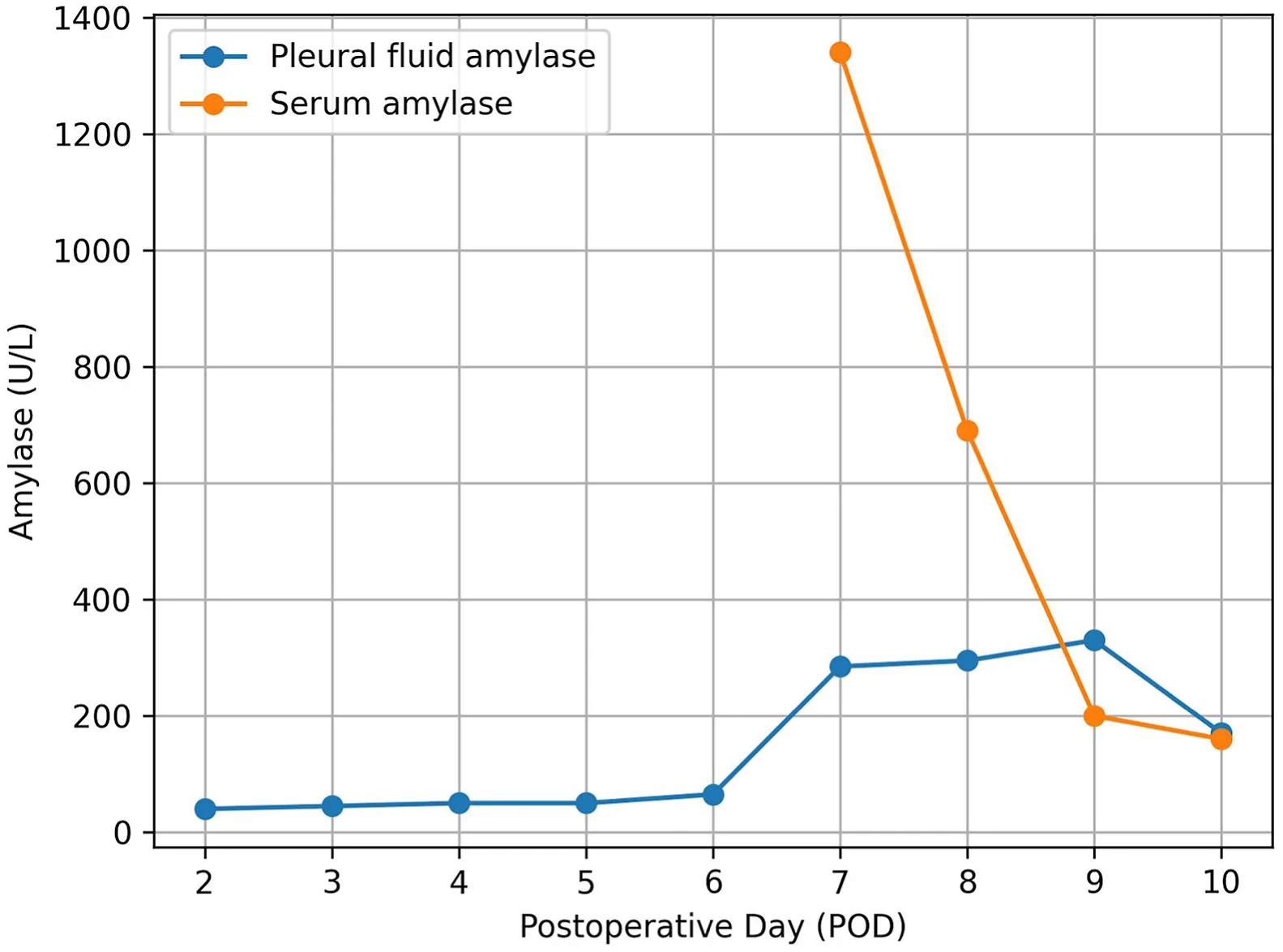
Serial drain fluid and serum amylase values.

## Comment

Pleural amylase monitoring is a sensitive, noninvasive, cost-effective, and practical screening tool for detecting anastomotic leak after esophagectomy.^1^, ^2^, ^3^, ^4^ In our institution, it plays an important role in early leak detection and has consistently proven to be clinically valuable. At the Lahey Clinic, we follow daily pleural amylase starting POD 2. A level of >200 U/L signals the need to further interrogate the anastomosis, although we find the pleural amylase level trend more informative than a specific cutoff value. Nevertheless, this case report highlights an important nuance in the interpretation of postoperative drain amylase. Not all postoperative amylase elevations originate from the anastomosis, particularly when the rise is only modest and the patient remains clinically reassuring. Acute pancreatitis, although uncommon in this setting, can elevate serum and pleural amylase and create the impression of an anastomotic leak.

Rather than diminishing the utility of postesophagectomy serial drain amylase monitoring, this case highlights the importance of contextual interpretation. When drain amylase rises unexpectedly in a patient who appears otherwise well without additional signs of anastomotic complication, clinicians should verify that the elevation is not a false positive driven by pancreatitis. Obtaining simultaneous serum amylase and lipase is a simple and effective step, which we have now integrated, that can distinguish pancreatic from anastomotic sources and prevent unnecessary interventions. Further evaluation by imaging or endoscopic methods is reserved for select cases.

Recognizing the rare false positive caused by pancreatitis allows surgeons to continue leveraging the excellent sensitivity of drain amylase without overdiagnosing anastomotic leak after esophagectomy. A simple confirmatory assessment when results are discordant with the clinical picture can preserve the reliability of this important postoperative diagnostic tool.
